# 387. Differences in frequency of *C. difficile* infection testing of inpatients with diarrhea at selected acute care hospitals in NY and GA, 2020.

**DOI:** 10.1093/ofid/ofac492.465

**Published:** 2022-12-15

**Authors:** Scott Fridkin, Christopher J Myers, Infectious Diseases, Udodirim N Onwubiko, William C Dube, Chad Robichaux, Sahil Khanna, Joann M Zamparo, Frederick J Angulo, Ghinwa Dumyati

**Affiliations:** Emory University School of Medicine, Atlanta, GA; University of Rochester, Rochester, New York; Emory University, MCDONOUGH, Georgia; Emory University, MCDONOUGH, Georgia; Emory University, MCDONOUGH, Georgia; Mayo Clinic, Rochester, Minnesota; Pfizer Vaccines, Killingworth, Connecticut; Pfizer Vaccines, Killingworth, Connecticut; University of Rochester Medical Center, Rochester, New York

## Abstract

**Background:**

*Clostridioides difficile* infection (CDI) incidence estimates vary between geographic regions; few studies have evaluated the impact of CDI test order frequency on estimated CDI incidence. We evaluated this impact in a sample of hospitals at two CDC Emerging Infections Program (EIP) sites.

**Methods:**

Daily surveillance was conducted for diarrhea among inpatients at 5 acute care hospitals (2,379 beds) in EIP sites in NY (2 hospitals) and GA (3 hospitals) during two 10-workday periods in 2020 and 2021. Diarrhea onset, test orders, and specimen collection status were ascertained. Stools were tested by PCR/NAAT initially or after negative EIA toxin. Differences in diarrhea incidence, testing frequency, and CDI positivity across site, care locations and hospitals were compared using Wilcoxon rank sum test. Correlates of CDI testing and positivity were assessed using modified Poisson regression. Estimates of incidence using EIP methodology at 5 hospitals was compared between sites using Mantel-Hanzel summary rate ratio.

**Results:**

Surveillance of 38,365 patient-days (PD) identified 860 diarrhea cases from 107 patient-care locations mapped to 26 unique NHSN defined location-types. Incidence of diarrhea was 22.4/1000 PD (medians 25.8 NY, 16.2 GA, P< 0.01); with similar proportions of diarrhea being hospital-onset (66%) and CDI positive (17%) by site. Overall, 35% were tested for CDI (21% NY, 49% GA, P< 0.01). Percent tested varied by NHSN location type (Figure). Regression models identified location-type (oncology, critical care), laxatives use, chemotherapy, and residing in EIP catchment area predictive of testing (Figure). Adjusting for these factors, NY was 49% less likely than GA to test (aRR 0.51, 95% CI 0.40-0.63). Simulation of EIP methods estimated NY had a 38% lower incidence of CDI than GA (summary rate ratio 0.62, 95% CI, 0.54-0.71).

**Figure:**
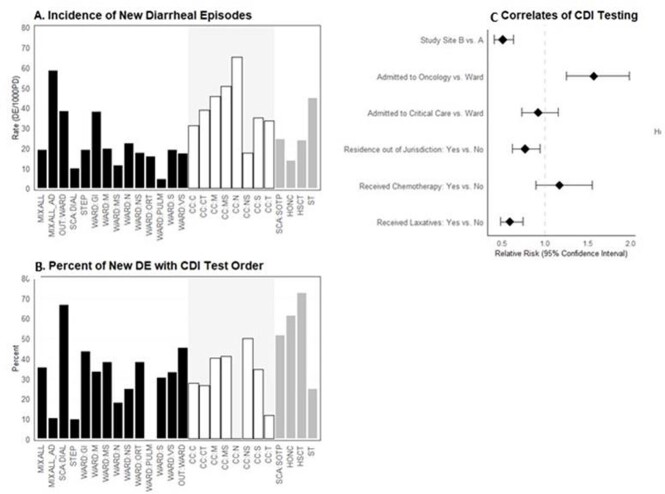

Incidence of Diarrheal Episodes (A) and Proportions Tested (B) among Hospitalized Patients, 2021 (solid, ward; open, critical care; grey, oncology), and adjusted relative risk (solid circles) with 95% confidence intervals (whisker) of independent predictors of testing for CDI (C).

**Conclusion:**

After adjusting for patient characteristics (e.g., location-type, laxative use), the likelihood of testing still differed between NY and GA sites; the magnitude of the differences in testing was similar to that observed in estimated CDI incidence. Testing practices likely influence surveillance data and is a consideration when comparing data across regions.

**Disclosures:**

**Scott Fridkin, MD**, Pfizer: Grant/Research Support **christopher J. Myers, MS, Infectious diseases**, Pfizer: Grant/Research Support **Udodirim N. Onwubiko, MBBS MPH**, Pfizer: Grant/Research Support **William C. Dube, MPH**, Pfizer: Grant/Research Support **Sahil Khanna, MBBS, MS**, Pfizer: Grant/Research Support **Joann M. Zamparo, MPH**, Pfizer: Employee|Pfizer: Stocks/Bonds **Frederick J. Angulo, DVM PhD**, Pfizer Vaccines: Employee|Pfizer Vaccines: Stocks/Bonds **Ghinwa Dumyati, MD**, Pfizer: Grant/Research Support.

